# Prevalence, characteristics and correlates of a positive-dementia screen in patients on antiretroviral therapy in Bamenda, Cameroon: a cross-sectional study

**DOI:** 10.1186/1471-2377-13-86

**Published:** 2013-07-15

**Authors:** Julius Atashili, Bradley N Gaynes, Brian W Pence, Gladys Tayong, Dmitry Kats, Julie K O’donnell, Peter M Ndumbe, Alfred K Njamnshi

**Affiliations:** 1 Department of Public Health and Hygiene, Faculty of Health Sciences, University of Buea, Rm 222 FHS building, 63 Molyko, Buea, Cameroon; 2 Department of Psychiatry, University of North Carolina School of Medicine, Chapel Hill, NC, USA; 3 Department of Community and Family Medicine, Duke University, Durham, NC, USA; 4 Center for Health Policy and Inequalities Research, Duke Global Health Institute, Duke University, Durham, NC, USA; 5 Bamenda Regional Hospital and North West Regional coordination of the National AIDS Control Committee, Bamenda, Cameroon; 6 Department of Epidemiology, Gillings School of Global Public Health, University of North Carolina, Chapel Hill, NC, USA; 7 Department of Biomedical Sciences, Faculty of Health Sciences, University of Buea, Buea, Cameroon; 8 Department of Neurology, Central Hospital Yaoundé/Faculty of Medicine and Biomedical Sciences, The University of Yaounde I, Yaounde, Cameroon

**Keywords:** HIV, Dementia, Screen, Cameroon, Antiretroviral therapy

## Abstract

**Background:**

In this study we assess the prevalence, characteristics as well as socio-demographic and clinical correlates of a positive screen for HIV-associated dementia in a group of patients on antiretroviral therapy (ART) in Bamenda, Cameroon.

**Methods:**

In a cross-sectional study, a structured questionnaire was used to collect data on 400 patients attending the Bamenda Regional Hospital AIDS-treatment Centre. Patients were assessed for neurocognitive function using the International HIV Dementia Scale (IHDS) to assess finger-tapping (FT), alternating hand sequence (AHS) and a 4-word recall (4WR), each scored on a maximum of four.

**Results:**

A total of 297 (74%) participants were females. The total IHDS score ranged from 6–12 with a mean of 9.02 and 85% of subjects screened positive for dementia (≤10 on IHDS). Participants performed worst in the AHS assessment with a mean of 2.25 (IQR: 2–3). In multivariable analyses, screening positive for dementia was significantly associated with having primary education or less (aOR: 8.33, 95%CI: 3.85, 16.67), and having HIV symptoms (aOR: 12.16, 95%CI: 3.08, 48.05).

**Conclusions:**

A very high proportion of patients on ART screened positive for dementia using the IHDS. This could potentially be an indication of a high prevalence of HIV-associated neurocognitive disorders in this population and or a poor performance of the IHDS in patients on ART. Future studies will need to assess the validity of the IHDS in this population of patients on ART and also evaluate long term outcomes in patients with positive dementia screens.

## Background

HIV infection often results in a spectrum of neurocognitive disorders, referred to as HIV-associated neurocognitive disorders (HAND), which need to be diagnosed and appropriately managed early [1,2]. While these disorders may result directly from the effects of HIV infection on the central nervous system, their clinical manifestation, and thus diagnosis, may be impacted by the overall clinical and neurological status of the patient as well as by the use of antiretroviral therapy (ART), some of which may have neurotoxic effects [3,4].

Systematic reviews of studies conducted worldwide suggest a prevalence of HAND ranging from 12-56% although the varying diagnostic tools used and study populations involved limit any direct comparisons and inferences to other study populations [5].

Little is known on the epidemiology of neurocognitive disorders in HIV in Cameroon. A study by Njamnshi et al. [6] reported a prevalence of 21.1% in a group of 204 HIV-positive patients in Yaounde. In a follow-up study, Kanmogne et al. using a battery of 19 standard neuropsychological measures in a cross-sectional study with 44 HIV-positive adults and 44 demographically matched HIV-negative controls, reported worse overall cognition in the HIV-positive individuals [7]. Furthermore, significantly lower performance was seen in the HIV-positive sample on tests of executive function, speed of information processing, working memory, and psychomotor speed. HIV-positive participants with AIDS performed worse than those with less advanced HIV disease [7]. Nevertheless, data on the epidemiology of neurocognitive disorders in HIV patients in general and patients on ART in particular remain scarce in this region of the world.

With the goal of providing epidemiological data that would be useful in designing guidelines for the care of mental diseases in HIV-affected patients in resource-limited settings, we assessed the prevalence, characteristics and demographical and clinical correlates of a positive dementia screen in a group of patients on antiretroviral therapy in Bamenda, Cameroon.

## Methods

### Ethical considerations

This study received ethical and administrative approval respectively from the Cameroon National Ethics Committee (No. 111/CNE/SE/09), the University of North Carolina at Chapel Hill’s Biomedical Institutional Review Board (# 09–0852) and the Duke University Health System IRB (# Pro00016937); and the Ministry of Public Health in Cameroon.

This manuscript was written following STROBE guidelines for the reporting of observational studies [8].

#### Study design and setting

We conducted a cross-sectional descriptive study as part of a wider project to assess and adapt the diagnosis and treatment of depression in HIV-positive patients in Bamenda, Cameroon- the ADEPT study. Details of the project have been described elsewhere [9,10]. In this paper we focus on dementia screening data as well as its correlates.

Briefly, the study was conducted in the Bamenda Regional Hospital AIDS Treatment Centre (BRHATC ) – a centre that receives close to 4000 patients per year for routine HIV-care including follow-up by a physician and paramedical staff.

#### Study population, sampling and participants

The study population was made of attendants of the BRHATC. To be eligible participants had to be aged between 18 and 55 years, be on antiretroviral therapy, speak English, and provide written informed consent. Severely ill patients, needing admission or not able to freely communicate were excluded.

Participants were enrolled in the period between May 2010 and October 2010. Each participant was eligible only once. In an attempt to get a sample representative of all attendees to the centre and because it was not possible to recruit and satisfactorily assess all attendees on a particular day (the daily attendance was estimated at more than 50), participants were approached consecutively as they registered at the beginning of their visit in the centre. Once a participant agreed to participate they were given the opportunity to undergo all study enrolment processes (consent and initial identification) after which the research assistant returned to the registration point to recruit the next available participant. Recruitment was pursued until the calculated sample size of 400 was reached.

#### Data collection, variables and measurements

Standardized questionnaires were used to collect data. The questionnaires, all administered by research assistants, included the following modules administered in chronological order: the Patient Health Questionnaire 9 (PHQ-9) to screen for depressive symptoms in the past two weeks [11]; a socio-demographic questionnaire (including an assessment of gender, age, marital status, education, and residence), a subjective assessment (as “fair” or “excellent”), by the research assistant, of participant’s English proficiency; a questionnaire assessing 13 HIV-related symptoms (new or persistent headaches, fever, oral pain, white patches in the mouth, rashes, nausea, trouble with eyes, sinus infection, numbness in the hands or feet, persistent cough, diarrhoea, weight loss, or abnormal vaginal discharge in women); the World Mental Health Survey Initiative Version of the World Health Organization’s Composite International Diagnostic Instrument CIDI (used to assess for lifetime depression and depression diagnosis within the preceding year) [12]; and the International HIV Dementia scale (IHDS). It is worth noting that it took at least 20 minutes of contact between research staff and study participant before the IHDS tool was administered, thus ensuring that participants were sufficiently relaxed and comfortable with the research staff before the IHDS assessments were conducted.

The IHDS is a standardized cross-culturally assessed tool. It was initially developed and assessed in participants from the USA and Uganda and shown to have sensitivities and specificities of 80% and 57% and 80% and 55% respectively using a cut-off of ≤10 [13]. The tool consists of three assessments: an assessment of motor speed through finger-tapping (FT); an assessment of psychomotor speed through a defined alternating hand sequence (AHS) that a participant is asked to repeat; and an assessment of memory recall through a 4-word recall (4WR) test. FT and AHS are scored on a five-point scale (0–4) while the 4WR is scored out of a maximum of four points, corresponding to the number of words correctly recalled. The IHDS has been used in other populations in sub-Saharan Africa, including in Cameroon [6]. We thus did not seek to re-assess its validity in our study population. However, to reduce the potential for bias in this study the assessment was made solely by a research assistant (a nurse by profession) trained by the study psychiatrist (BNG). The four words “Dog”, “Hat”, “Bean”, and “Red” were used in the 4WR assessment.

#### Data management and statistical analysis

Data from the questionnaire were entered into an Epi-Info 2000 database (WHO/CDC Atlanta, USA) and analysed using STATA 10 (STATA corps, Dallas, TX, USA). Participant characteristics were described using means, medians, standard deviations and interquartile ranges (IQRs) for continuous variables, and using absolute and relative frequencies of various responses for categorical variables.

To characterize neurocognitive function, we described the frequency of each possible response to each of the three assessments (FT, AHS, 4WR). We also computed the mean, median, range and IQR of the scores for each of the three assessments.

The composite score on the IHDS was computed by adding the scores from the three individual assessments. Correlates of a positive dementia screen were assessed using two methods: considering the IHDS score on a continuous scale (a lower score indicating more impaired neurocognitive function) and considering it as a binary measure with a cut-off point of 10 as used in other settings (a score less than or equal to 10 being considered a positive dementia screen) [13]. In considering the IHDS score as a continuous variable, the bivariable analysis consisted of computing the mean difference (and 95% confidence interval) in the aggregate scores between groups of participants while the multivariable analysis consisted of determining the adjusted mean difference between groups using a multiple linear regression model with IHDS score as outcome and patient characteristics considered as predictors. Predictors were assessed for linearity before being included in the regression model. Considering the IHDS score as a binary measure, the bivariable analysis consisted of comparing the odds of having a positive screen between groups of participants using a Mantel-Haensel Odds ratio (and 95% CI) while the multivariable analysis consisted of computing adjusted odds ratios (aOR) using a multiple logistic regression model with IHDS positive screen as the outcome and patient characteristics considered as predictors.

A sensitivity analysis was conducted to assess the potential impact (on prevalence and correlates) of using an IHDS score cut-off point of ≤9 in defining a positive screen.

A sample size of 400 participants allowed us to estimate a positive dementia screen prevalence of 50%, at a 95% confidence level, allowing for a margin of error of ±5% and a non-response rate of 5%.

## Results

### Study participants

The characteristics of the 400 participants included in this analysis are presented in Table 1. A vast majority were females (74%) and self-declared Christians (99%). The median age was 41 years (IQR 34–47) and 85 (21%) had never been married. One hundred and fifty-five (39%) had greater than primary level education with the English competency being assessed as excellent in 40%. Two-hundred and forty-four participants (61%) declared living in an urban area and the estimated median daily expenditure was one US dollar (IQR 1–3).

**Table 1:** Characteristics of the overall study population (N (%) or median (IQR))

**Characteristic**	**N or Median**	**% or IQR**
Sex		
Male	103	26
Female	297	74
Age	41	34-47
Religion		
Christian	394	99
Other	5	1
Marital status		
Married/cohabitating	137	34
Previously married	178	44
Never married	85	21
Education*		
Primary	245	61
Greater than primary	155	39
Daily expenditures**	1	1-3
Village of residence		
Urban	244	61
Rural	156	39
Competency in English		
Excellent	158	40
Fair	242	60
HIV symptom score (possible range: 0–13)	5	3-6
Dementia		
Screen positive, cut-off = 10	333	85
Screen positive, cut-off = 9	235	59

*Primary = 6 years or fewer; greater than primary = more than 6 years.

**In US dollars, approximation based on reported weekly expenditures in FCFA. IQR: Interquartile range.

#### Overall IHDS score and prevalence of a positive dementia screen

The total IHDS score ranged from 6–12 with an IQR of 8–10 and a mean score of 9.02. The prevalence of participants with a positive screen was respectively 85% and 59% when cut-offs of ≤10 and ≤9 were used.

#### Characteristics of dementia screen

Of the three assessments, participants performed worst in the AHS assessment (Table 2). The mean score in the AHS assessment was 2.25 with an IQR: 2–3. Most participants (62%) could complete only two alternating hand sequences in 10 seconds. The performance in the 4WR assessment was relatively better with a mean score of 3.28 and an IQR of 3–4. Only 46% could recall all four words in the 4WR assessment. The best performance was obtained in the FT assessment with a mean score of 3.49 and an IQR of 3–4. Approximately half (51%) of participants could complete 15 or more finger taps in 5 seconds and only 2% completed 10 or fewer finger taps in 5 seconds.

**Table 2:** Breakdown of International HIV Dementia Scale (IHDS) Scores

	**Mean (SD)**	**Range (IQR)**	**N (%)**
IHDS total score	9.02 (1.39)	6-12 (8–10)	
IHDS finger-tapping subscore	3.49 (0.53)	2-4 (3–4)	
15 taps in 5 seconds			202 (51)
11-14 taps in 5 seconds			191 (48)
7-10 taps in 5 seconds			6 (2)
3-6 taps in 5 seconds			
0-2 taps in 5 seconds			
IHDS alternating hand sequence	2.25 (0.57)	1-4 (2–3)	
4 sequences in 10 seconds			1 (0.25)
3 sequences in 10 seconds			125 (31)
2 sequences in 10 seconds			246 (62)
1 sequences in 10 seconds			27 (7)
0 sequences in 10 seconds			
Unable to perform			
IHDS 4-word recall	3.28 (0.77)	1-4 (3–4)	
4 words recalled			182 (46)
3 words recalled			152 (38)
2 words recalled			58 (15)
1 word recalled			7 (2)

Abbreviations: *IHDS* International HIV Dementia Scale, *IQR* Interquartile range, *SD* Standard deviation.

#### Correlates of a positive dementia screen

The socio-demographic correlates of a positive dementia screen are presented in Table 3 and Table 4. In bivariable analyses with a cut-off of ≤10, the factors that appeared to be associated with a positive screen included being of female gender, increasing age, having only primary education (compared to greater than primary education), being previously married or currently married/co-habiting (when compared to having never been married), having a diagnosis of depression within the past year, and having symptoms associated with HIV-infection (Table 3). However, after adjusting for potential confounding by each of the previously cited factors, only marital status, education and having symptoms associated with HIV-infection remained significant predictors of a positive dementia screen (Table 4).

**Table 3:** Correlates of dementia bivariable analysis

	**Positive dementia screen (≤10 on IHDS)**		**IHDS continuous scale**	
	**OR**	**95**% **CI**	**Mean difference**	**95**% **CI**
Gender				
Male	REF		REF	
Female	2.16	1.24, 3.77	−0.68	−0.99, -0.3
Age				
20–29	REF		REF	REF
30–39	2.44	1.17, 5.08	−0.35	−0.78, 0.07
40–49	3.75	1.75, 8.08	−0.89	−1.31, -0.46
50+	12.09	3.29, 44.43	−1.67	−2.15, -1.19
Marital Status				
Married/co-habitating	REF		REF	
Previously married	3.22	1.56, 6.65	−0.64	−0.93, -0.35
Never married	0.48	0.26, 0.89	0.66	0.31, 1.01
Education				
Primary	REF		REF	
Greater than primary	0.10	0.05, 0.19	1.52	1.29, 1.76
Depression diagnosis				
Past month	0.52	0.13, 2.01	0.45	−0.38, 1.28
Past 6 months	0.44	0.16, 1.19	0.67	0.04, 1.29
Past year	0.41	0.18, 0.94	0.69	0.17, 1.21
Lifetime	0.89	0.47, 1.68	−0.01	−0.34, 0.33
Number of lifetime depressive episodes				
0	REF		REF	
1	1.25	0.53, 2.95	0.004	−0.41, 0.41
2+	0.73	0.38, 1.38	0.11	−0.24, 0.46
HIV Symptoms				
0	REF		REF	
1–4	5.60	1.79, 18.58	−1.08	−1.84, -0.32
5–9	16.00	4.68, 54.73	−1.52	−2.28, -0.76
10+	19.20	3.10, 119.05	−1.88	−2.78, -0.99
HIV Symptoms				
0	REF		REF	
>0	9.05	2.86, 28.62	−1.33	−2.09, -0.58
HIV Symptom Score*	1.28	1.14, 1.44	−0.11	−0.16, -0.07
Village of residence				
Urban	REF		REF	
Rural	1.46	0.83, 2.56	−0.30	−0.58, -0.02

Abbreviations: *For each additional symptom; *CI* Confidence interval, *IHDS* International HIV Dementia Scale, *IQR* Interquartile range, *REF* Reference category, *SD* Standard deviation.

**Table 4:** Correlates of dementia – multivariable analysis

	**Positive dementia screen (≤10 on IHDS)**		**IHDS Continuous scale**	
	**Adjusted OR**	**95**% **CI**	**Adjusted mean difference**	**95**% **CI**
Gender				
Male	REF		REF	
Female	1.92	0.92, 3.99	−0.48	−0.75, -0.22
Age				
20–29	REF		REF	
30–39	2.05	0.86, 4.89	−0.17	−0.53, 0.19
40–49	2.43	0.91, 6.49	−0.48	−0.86, -0.11
50+	4.20	0.93, 19.02	−0.94	−1.38, -0.51
Marital Status				
Married/co-habitating	REF		REF	
Previously married	2.56	1.06, 6.18	−0.31	−0.57, -0.06
Never married	0.73	0.34, 1.56	0.25	−0.07, 0.56
Education				
Primary	REF		REF	
Greater than primary	0.12	0.06, 0.26	1.1	0.91, 1.38
Depression diagnosis				
Past year	0.49	0.17, 1.41	0.37	−0.04, 0.78
HIV Symptoms				
0	REF		REF	
>0	12.16	3.08, 48.05	−0.97	−1.59, -0.35
Village of residence				
Urban	REF		REF	
Rural	1.22	0.63, 2.36	−0.14	−0.35, 0.08

Abbreviations: *CI* Confidence interval, *IHDS* International HIV Dementia Scale, *IQR* Interquartile range, *REF* Reference category, *SD* Standard deviation.

After adjustments, the odds of having a positive screen in previously married participants was 2.56 times (95%CI: 1.06, 6.18) that in currently married/co-habiting participants. Also, the odds of a positive screen in participants with greater than primary level education was 0.12 times (95%CI: 0.06, 0.26) that in participants with only primary education. The strongest predictor of a positive screen was the presence of symptoms associated with HIV-infection: after adjustment, the odds of having a positive screen in symptomatic participants was 12.16 times (95% CI: 3.08, 48.05) that in asymptomatic participants.

## Discussion

In this study we report a high prevalence of a positive screen for dementia using the IHDS with more than half of participants on ART in Bamenda screening positive. We also show that the primary function that appears impaired, based on this screening tool, is psychomotor speed while motor speed and memory recall are relatively better preserved. Few predictors of poor neurocognitive function were identified, with HIV-symptomatology being the strongest and the others being marital status and level of education.

To the best of our knowledge this is one of the few studies documenting and characterising the type of neurocognitive disorders in patients already on antiretrovirals in sub-Saharan Africa. Our positive screen prevalence estimate is very much higher than the 21% reported by Njamnshi et al., in HIV-positive patients in Yaounde, Cameron [6]. Our estimate is however very similar to the 80% reported by Robbins et al., in a population of patients on ART in South Africa [14]. Other studies conducted in Africa indicated lower screen positive rates: 22% in ART naïve patients in Lusaka, Zambia [15]; 14% in a mix of ART-naïve and experienced patients in Blantyre, Malawi (13.4% in patients on ART for at least six months) [16]; 38% in HIV-positive patients in Gaborone, Botswana (97.5% of whom were ART-experienced) [17].

It is not immediately clear to us why such a high positive screen rate was recorded. However, amongst others, we suspect that this could be an indication of a truly high prevalence of neurocognitive disorders in these patients who were already on antiretroviral therapy, an indication of relatively advanced HIV-disease. It is worthwhile noting that in this setting antiretroviral therapy was only recommended in patients with CD4+ counts less than 200 or having an AIDS-defining illness (WHO stage IV).

The high screen positive rate could also potentially be due to the IHDS performing poorly (with a low specificity thus high false positivity rate) in this population. This poor performance could be linked to the use of antiretroviral therapy some of which are known to cause peripheral neuropathy which could potentially reduce psychomotor speed. It is worthwhile noting that a vast majority of the patients in this clinic were on non-nucleotide reverse transcriptase inhibitor (NNRTI)-based antiretroviral therapy, with the NNRTI being combined with two nucleotide-based reverse transcriptase inhibitors (NRTI). Zidovudine and lamivudine are the most commonly used NRTI and have been shown to have neurologic side-effects that include peripheral neuropathy [4]. Efavirenz is also commonly used and has been shown to have CNS effects [4].

The poor performance by the IHDS could also be linked to the education status of participants. In an analysis of the performance of IHDS, Waldrop-Valverde et al., showed that only education was significantly associated with performance [18]. Further, a study by Nitrini et al., showed that performance on the AHS was inversely correlated with the number of years of schooling [19]. We note however that Sacktor et al. found no effect of educational level on the performance of the IHDS [13].

Though widely used, further studies are needed to validate the IHDS particularly in a large sample of patients on antiretroviral therapy and moderate to low education status [18,20,21]. This validation is particularly important as other authors have reported that certain screening tests may not be appropriate in certain populations. For example Robbins et al. in their assessment of the utility of the Montreal Cognitive Assessment scale reported that it may need to be modified before being used in South Africa [22].

Few studies have examined correlates of neurocognitive disorders in patients on ART. Lawler et al. [17] identified level of education and age (and not CD4 count) as significant correlates of disorders in patients in Botswana. Patel et al. [16]identified male gender and education level as risk factors associated with neurocognitive disorders. Joska et al. also confirmed older age and low education level as factors associated with HAND in South Africa [23]. Njamnshi et al. [24] reported advanced clinical diseases, low CD4 counts and low haemoglobin levels as risk factors for HAND in HIV-positive subjects in Yaounde, Cameroon. The latter findings are consistent with our findings of positive screen being correlated with increased HIV-symptomatology, indicative of relatively advanced disease.

While validity of our findings is vouched, amongst others, by the use of a well-trained health personnel in administering the IHDS, the use of a calm and re-assuring milieu while assessing the participants, the adequately large sample size of participants on antiretroviral therapy, and controlling for some confounding in the assessment of correlates, a number of limitations need to be considered in interpreting these data. First, the study is based on a screening tool, not a diagnostic tool – we did not systematically perform a diagnostic assessment and some of the positive screens could effectively be false positives. This limitation leads one to argue for a better assessment of not only the validity of the IHDS as a tool for screening for dementia in this population, but also for further prospective studies that evaluate the long-term evolution of patients who at some point screen positive for HAND. Only then will it be possible to make a recommendation on the utility, or lack thereof, of systematically screening in this population using the IHDS.

Other limitations include the lack of detailed information on HIV-disease such as CD4+ cell count levels and HIV-viral load at the time of study enrolment. However, the fact that these patients were on antiretroviral therapy and the high prevalence of symptoms suggest that the participants must have had advanced HIV-disease. The findings are certainly only representative of patients on antiretroviral therapy attending HIV-clinics like that of the Bamenda Regional Hospital. Some potential predictors could also have been missed as not all characteristics were assessed. The latter could also imply a limit on the adjustment for unmeasured confounders such as HIV-viral load.

## Conclusion

If confirmed, our findings suggest that neurocognitive disorders may be a significant concern in HIV-infected patients in settings such as ours. These disorders appear more prominent in patients with symptomatic diseases. Nevertheless further studies assessing the validity of the IHDS compared to a more specific gold-standard, and prospective studies describing the long term outcomes of a positive screen, as well as the effectiveness of early interventions on these outcomes will be needed for the development of guidelines specific to this population.

## Abbreviations

4WR: 4-word recall; AHS: Alternating hand sequence; AIDS: Acquired Immunedeficiency Syndrome; aOR: Adjusted odds ratio; ART: Antiretroviral therapy; BRHATC: Bamenda Regional Hospital AIDS treatment centre; CI: Confidence interval; CIDI: Composite international diagnostic instrument; CNS: Central nervous system; FT: Finger-tapping; HAND: HIV-Associated neurocognitive disorders; HIV: Human immunodeficiency virus; IHDS: International HIV dementia scale; IQR: Interquartile range; IRB: Institutional review board; NNRTI: Non-nucleotide reverse transcriptase inhibitor; NRTI: Nucleotide reverse transcriptase inhibitor; OR: Odds ratio; PHQ-9: Patient health questionnaire 9; STROBE: Strengthening the reporting of observational studies in epidemiology.

## Competing interests

The authors declare that they have no competing interests.

## Authors’ contributions

JA, BNG, BWP, PMN and AKN defined the question and designed the study. JA, BNG, BWP, GT, PMN and AKN supervised data collection and review. JKO and BWP conducted the statistical analysis. JA, BNG, BWP, DK, JKO interpreted the original results. All authors wrote or reviewed and approved the final manuscript.

## Pre-publication history

The pre-publication history for this paper can be accessed here:

http://www.biomedcentral.com/1471-2377/13/86/prepub
